# Efficacy and safety of different doses of ligelizumab in patients with chronic spontaneous urticaria: a systematic review and meta-analysis of randomized controlled trials with GRADE evaluation

**DOI:** 10.1007/s00210-025-04775-8

**Published:** 2025-11-18

**Authors:** Lamees Taman, Mohamed Abo Zeid, Kareem Khalefa, Habiba Tariq Saeed, Amr Alaa Azzouz Elkelany, Habiba Abdelhameed Elrefaey, Amr M. Abou Elezz

**Affiliations:** Faculty of Medicine, Tanta University, Tanta, Egypt

**Keywords:** Ligelizumab, CSU, ISS7, HSS7, UAS, Meta-Analysis

## Abstract

**Background:**

Chronic spontaneous urticaria (CSU) is a skin condition characterized by itchy hives and/or angioedema, impairing patients’ quality of life even with the current available medications. This study aims to evaluate the efficacy and safety of Ligelizumab, a high-affinity monoclonal anti-IgE antibody, in the treatment of CSU.

**Methods:**

We performed a systematic review and meta-analysis in accordance with PRISMA criteria, methodically exploring databases for randomized controlled trials (RCTs) comparing Ligelizumab with placebo in patients with CSU. Studies that reported outcomes like the itch severity score (ISS7), the hives severity score (HSS7), and adverse events were included in our analysis.

**Results:**

Four RCTs were included in our analysis with a total of 2,481 patients with CSU. Ligelizumab significantly improved urticaria symptoms as measured by change in ISS7 at week 12 in adults (MD = -3.67, 95% CI: [-4.25 to -3.09], p < 0.00001), change in UAS7 at week 12 in adults (MD = -9.18, 95% CI: [-10.45 to -7.92], p < 0.00001), and change in UAS7 at week 12 in adolescents (MD = -6.48, 95% CI: [-12.84 to -0.12], *p* = 0.05). Regarding safety outcomes, only Ligelizumab 120mg showed significantly higher upper respiratory tract infection rates (RR = 2.70, 95% CI [1.17 to 6.21], *p* = 0.02).

**Conclusion:**

Our findings suggest that Ligelizumab, at doses 72 and 120 mg, might be able to improve the symptoms of CSU evidenced by the improvements in ISS7 UAS7 and UAS7 response rate and the overall quality of life with an acceptable safety profile with certain concerns regarding Ligelizumab 120 mg causing upper respiratory tract infections. However, the small number of studies included limit the generalizability of our results.

**Supplementary Information:**

The online version contains supplementary material available at 10.1007/s00210-025-04775-8.

## Introduction

Chronic spontaneous urticaria (CSU) is a condition characterized by recurrent hives and intense itching lasting for more than six weeks. About 40% of people with chronic urticaria may experience episodes of angioedema, which is characterized by an abrupt enlargement of the deep dermis in well-defined regions such as the lips, periorbital region, extremities, and genitalia(1–3).

Typically, 70–95% of individuals have urticaria with unclear origin, a complex interaction of immunological, genetic, environmental, and psychological variables is believed to be responsible. Autoimmunity is a major contributing cause, in which autoantibodies attach to the high-affinity IgE receptor on mast cells or basophils, activating them and causing the production of histamine and different cytokines, which are critical mediators in CSU. Certain genetic variants associated to the immune system may increase sensitivity, suggesting that genetic predisposition may play a role (4–6).

The risk of CSU increases in middle aged women particularly those with certain medical conditions as thyroid diseases, infections as helicobacter pylori and allergic disorders. Other aggravating factors include psychological stress, food additives and certain medications as non-steroidal anti-inflammatory drugs (NSAIDs) and angiotensin converting enzyme inhibitors (ACEIs).(7,8) CSU causes significant psychosocial burden on patients leading to anxiety, depression and poor quality of life as persistent pruritus often leads to sleep disturbance, reduced work productivity and secondary skin infections.(9).

Currently licensed medications for CSU treatment include second-generation H1-antihistamines and anti-IgE Omalizumab. They block the production of histamine, reduce or eliminate hives and help ease symptoms of itch and swelling, they are better than first-generation antihistamines because they reduce the likelihood of sedation and anticholinergic side effects (10,11). Other treatment options for patients who are resistant to antihistamines include leukotriene-receptor antagonists, systemic glucocorticoids, cyclosporine, hydroxychloroquine, dapsone, methotrexate, sulfasalazine, and intravenous immune globulin. Studies have shown that Omalizumab, which is a recombinant humanized monoclonal antibody, may suppress allergen-mediated skin reactions through its reduction of Fc region of IgE (FcεRI) function in basophils and mast cells (10). Despite effectiveness of omalizumab in many patients, it has several limitations and reports lack of efficacy for some patients and specific disease conditions due to its moderate affinity for IgE (11). For example, it showed limited efficacy in atopic dermatitis and in patients with very low or very high IgE levels. Although most studies reported that omalizumab is well tolerated, adverse effects such as skin reactions, angioedema, headache, nausea, arthralgia, cough, fatigue, myalgia and rare cases of anaphylaxis were documented.(14–17).

Ligelizumab (QGE031) is a highly potent humanized IgG1 monoclonal antibody that targets and modulates the IgE pathway, a central mechanism in CSU pathogenesis. It acts by binding to IgE receptors at a different epitope than omalizumab, demonstrating a significantly higher affinity for IgE in vitro (approximately 50 times greater). This superior affinity translates to six-fold to nine-fold greater potency in vivo for suppressing free IgE.(18) Ligelizumab effectively blocks the interaction of free IgE with both its high-affinity receptor (FcεRI) on mast cells and basophils, and its low-affinity receptor (FcεRII/CD23). Treatment with Ligelizumab leads to rapid, dose- and time-dependent suppression of free IgE, even in patients with high baseline IgE levels and a subsequent reduction in FcεRI and IgE expression on circulating basophils. These actions collectively result in a significant improvement of allergic responses.(18,19).

In this study, we are investigating the efficacy and safety of different doses of Ligelizumab in the treatment of CSU through a systematic review and meta-analysis of randomized clinical trials.

## Methods

In this systematic review and meta- analysis, we followed PRISMA (Preferred Reporting Items for Systematic Reviews and Meta-Analyses) guidelines (20). The GRADE approach was used to assess the strength of the evidence. We registered the protocol of this study Open Science Framework (OSF) (DOI: 10.17605/OSF.IO/5HUYX).

### Search strategy and data sources

We searched PubMed, Web of Science, Cochrane, and Scopus for relevant studies up to 21 st December 2024. For a meticulous search strategy, we used the MeSH keywords “Ligelizumab.” In combination with “chronic spontaneous urticaria.”

The search strategy for these databases was: (Ligelizumab OR “QGE-031” OR “QGE031”) AND (“Chronic spontaneous urticaria” OR “Chronic idiopathic urticaria” OR “CSU” OR “CIU” OR “Chronic hives” OR “Recurrent hives” OR “Chronic urticaria” OR “Idiopathic urticaria” OR “Spontaneous urticaria” OR urticaria).

### Selection criteria

We included studies that met the following criteria: 1) Randomized clinical trials (RCTs) which compared Ligelizumab at any dose with placebo including patients with CSU. 2) Studies that reported at least one of the following outcomes: Hives severity score (HSS7), Itch severity score (ISS7), urticaria activity score (UAS7).

We excluded studies that met any of the following criteria:—1) case-reports, animal studies, reviews, editorials. 2) Studies with only abstract or unavailable full text or overlapped data. 3) Studies with other interventions or without a comparison group.

### Study selection

The findings of the literature search were screened by two independent, blinded authors using Rayyan software on two stages. (21) Firstly, the abstracts and titles of potential studies were screened, then, the full text of the remaining studies were screened for their eligibility. Any conflicts were resolved by the senior author.

### Data extraction

We extracted the data from each included study using an online data extraction sheet. The extracted data included four domains:—1) study characteristics, 2) Baseline and Demographics of the include patients (age, sex, race, BMI), 3) Efficacy outcomes (Change From Baseline in HSS7, ISS7, UAS7 and finally UAS7 Response), 4) safety outcomes (Patients with at least 1 adverse events (AEs), Cardiovascular and Cerebrovascular events, Headache, Upper respiratory tract infection and Hypersensitivity reactions).

### Quality assessment

We evaluated the RCTs’ methodological quality using the Cochrane risk-of-bias tool for randomized trials (RoB-2) (22), which assesses six important domains: (1) bias in the selection of reported results; (2) bias resulting from deviations from intended interventions; (3) bias resulting from missing outcome data; (4) bias in outcome measurement; (5) bias in the selection of reported results; and (6) overall bias. Every element is classified as “low risk,” “some concern,” or “high risk.” Any conflicts were resolved with a third author.

### Data synthesis and statistical analysis

Data analysis was conducted using RevMan Cochrane software, (version 5.4) (23). Dichotomous data (headache, upper respiratory tract infections, hypersensitivity reactions) were analyzed using risk ratio (RR) while continuous data (ISS7, HSS7, UAS7) were analyzed using mean difference (MD) with a 95% confidence interval (CI) and P-values < 0.05 were considered statistically significant for all outcomes.

### Assessment of heterogeneity

Heterogeneity was assessed using the Chi-Square (χ2) and I2 tests. Heterogeneity was considered significant when the chi-square test p-value is less than 0.1 and the I^2^ test is more than 50%. We conducted subgroup analyses according to different doses of Ligelizumab.

## Results

### Search results and study selection

**Fig. 1:**
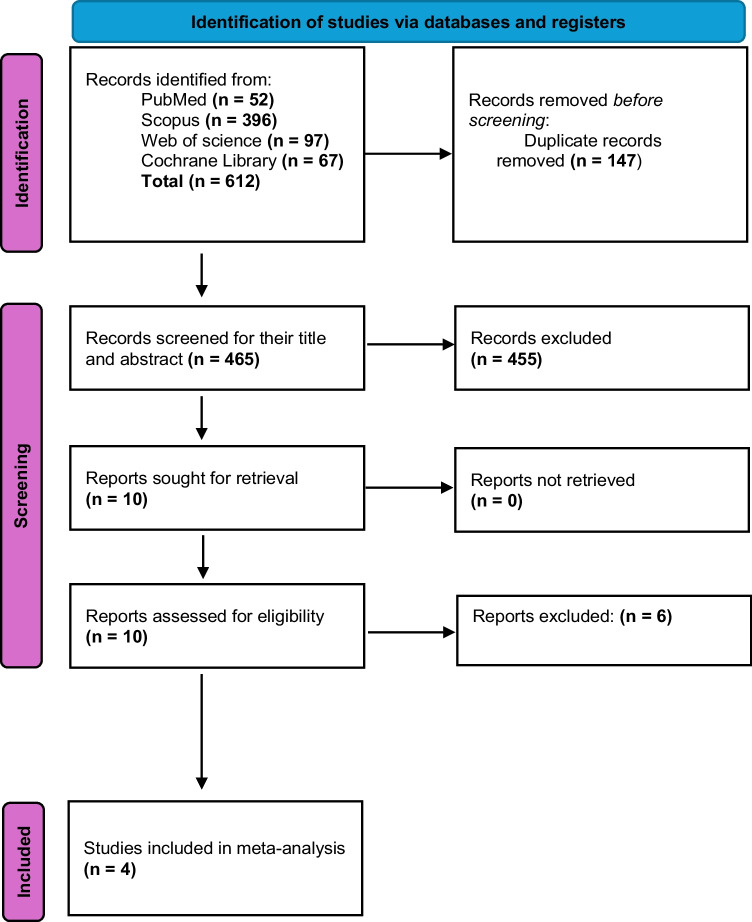
PRISMA Flowchart

A total of 612 studies were initially identified by using the predefined search strategy on the different databases (PubMed, Scopus, Web of Science and Cochrane library), 147 of which were duplicated and removed. On screening the titles and abstracts of the remaining 465 studies using the eligibility criteria mentioned above, 455 articles were found irrelevant. When evaluating the full text of the remaining ten articles, only four studies were included in our study. **(**Fig. 1**).**

### Characteristics of included studies

Four RCTs were included in our analysis with a total of 2,574 patients suffering from CSU (2,432 adults and 142 adolescents). Patients were randomized to receive either different doses of Ligelizumab (24, 72, 120, 240, or 300 mg) or placebo **(**Table 1**)**. The mean age of adult participants ranged from 14.3 years to 41.8 years while in adolescents it ranged from 14.3 to 15.9 years, and the BMI ranged from 22.2 to 25.8 kg/m^2^ (Table 2**).**

**Table 1:** Summary of the included studies

Study ID (no. of centers)	Study Design	Study arms			No. of participants	Follow up duration	Main inclusion criteria
		Intervention arm		Control arm			
		Drug	Dose				
PEARL-1 (28 centers) PEARL-2 (27 centers)	RCT	Ligelizumab	72 mg	Placebo (+ 120 mg ligelizumab from week 24 onwards)	**PEARL-1** 1030 adults 38 adolescences **PEARL-2** 1020 adults 55 adolescences	64 weeks	1.Male and female participants aged 12 years or older at the time of screening 2.Diagnosis of chronic spontaneous urticaria (CSU) for at least 6 months 3.CSU that is refractory to H1-antihistamines (H1-AH) at approved doses as confirmed by the following criteria: 4.Persistent itch and hives for at least 6 consecutive weeks at any time prior to Visit 1 (Day −28 to Day −14), despite current use of non-sedating H1-antihistamines 5. A Urticaria Activity Score over 7 days (UAS7) of 16 or higher (scale: 0–42) and a Hives Severity Score over 7 days (HSS7) of 8 or higher (scale: 0–21) during the 7 days preceding randomization (Visit 110, Day 1) 6.Use of H1-antihistamines at locally approved doses for CSU treatment beginning from Visit 1 (Day −28 to Day −14) 7.Willingness and ability to consistently complete a daily symptom eDiary throughout the study and adhere to the study visit schedule
		Ligelizumab	120 mg				
		Omalizumab	300 mg				
Staubach 2022 (20 centers)	RCT	Ligelizumab	24 mg	Placebo (till week 12 then shifted to 120 mg ligelizumab)	49 adolescents	40 weeks	1- Adolescent patients (≥ 12 years and < 18 years) with CSU (for > 6 months) 2- Experiencing itch and hives for ≥ 6 consecutive weeks despite using approved doses of nonsedating H1-AH 3- Weekly Urticaria Activity Score (UAS7) ≥ 16 and weekly Hives Severity Score (HSS7) ≥ 8 during 7 days prior to randomization
		Ligelizumab	120 mg				
Maurer 2019 (82 centers)	RCT	Ligelizumab	24 mg	Placebo	382 adults	24 weeks	1- Diagnosis of chronic spontaneous urticaria for at least 6 months 2- Refractory to standard of care at time of randomization
		Ligelizumab	72 mg				
		Ligelizumab	240 mg				
		Omalizumab	300 mg				
		Ligerlizumab	120 mg (single dose)				

UAS7: Urticaria Activity Score over 7 days**; **HSS7**: **Hives Severity Score over 7 days**; **CSU**: **chronic spontaneous urticaria**; **ISS7**:**Itch severity score over 7 days; RCT**: **Randomized controlled trials

**Table 2:** Demographics and baseline characteristics of patients in the included studies

Study ID	Study arms	Age (years), mean (SD) adults	Age (years), mean (SD) adolescents	BMI (kg/m2), mean (SD)	Sex (female), N (%) adults	Sex (female), N (%) adolescent	Total IgE mean (SD)	UAS7 mean (SD)	ISS7 mean (SD)	HSS7, mean (SD)	Duration chronic spontaneous urticaria (CSU), (years), mean (SD)	Race, N (%) adults				Race, N (%) adolescent			
												Black	White	Asian	Other	Black	White	Asian	Other
PEARL-1	**72 mg LIG**	42·3 (13·5)	15.1(1.62)	27·6 (5·9)	217 (71%)	9 (75.0%)	109.6 (125.95)	29·84 (7·2)	13·41 (4·0)	16·43 (4·3)	2.9(3.50)	1 (< 1%)	226 (74%)	60 (20%)	20 (7%)	1 (8.3%)	8 (66.7%)	3 (25%)	0
	**120 mg LIG**	43·3 (13·1)	14.6(2.01)	27·2 (5·7)	226 (72%)	5 (50.0%)	128.1(157.2)	30·08 (7·3)	13·46 (4·3)	16·62 (4·3)	2.17(2.23)	4 (1%)	220 (71%)	72 (23%)	16 (5%)	0	8 (80%)	0	0
	**300 mg OMA**	42·2 (13·2)	14.7(1.65)	27·7 (6·4)	218 (71%)	9 (69.2%)	127.8(156.2)	29·55 (7·7)	13·20 (4·5)	16·35 (4·5)	3.23(4.096)	9 (3%)	221 (72%)	64 (21%)	15 (5%)	0	8 (61.5%)	2(15.4%)	0
	**Placebo**	43·2 (14·1)	15.3(2.08)	27·9 (5·2)	76 (72%)	1(33.3%)	113.6(133.4)	30·87 (7·3)	13·89 (4·2)	16·98 (4·2)	3.33(4.06)	1 (1%)	80 (75%)	22 (21%)	3 (3%)	0	2(66.7%)	0	0
PEARL-2	**72 mg LIG**	41·7 (13·4)	14.3(1.39)	27·8 (5·7)	227 (74%)	10 62.5%	105.4(122.1)	29·37 (8·0)	13·10 (4·7)	16·26 (4·6)	2.9(3.72)	5 (2%)	227 (74%)	66 (21%)	9 (3%)	1(6.25%)	9 (56.25%)	2 (12.5%)	0
	**120 mg LIG**	43·0 (14·1)	15.1(1.56)	27·3 (5·8)	211 (69%)	12 63.2%	135.1(159.2)	29·54 (7·7)	13·31 (4·6)	16·23 (4·5)	2.9(3.58)	12 (4%)	218 (72%)	67 (22%)	7 (2%)	0	15(78.94%)	2(10.53%)	0
	**300 mg OMA**	44·1 (14·1)	15.9(1.17)	27·8 (6·7)	225 (73%)	10 71.4%	145.2(151.5)	30·12 (7·8)	13·48 (4·7)	16·64 (4·3)	2.7(2.28)	9 (3%)	228 (74%)	63 (20%)	9 (3%)	0	10(71.42%)	4 (28.57%)	0
	**Placebo**	42·9 (13·0)	14.8(1.47)	26·5 (5·3)	80 (78%)	4 66.7%	121.3(144)	31·10 (6·7)	13·65 (4·4)	17·45 (3·8)	3(3.53)	1 (1%)	79 (77%)	19 (18%)	4 (4%)	0	5(83.3%)	0	0
**Staubach 2022**	**24 mg LIG**	NA	14.9 (1.9)	23.9 (6.5)	NA	10 (41.7%)	194.4(92)	30.5 (7.3)	14.7 (4.8)	15.8 (4.0)	2.0 (1.5)	NA	NA	NA	NA	1 (4.2%)	16 (66.7%)	7 (29.2%)	NA
	**120 mg LIG**	NA	15.2 (1.4)	22.2 (3.9)	NA	9 (69.2%)	202.4(195)	29.3 (7.7)	12.8 (4.5)	16.5 (4.6)	3.8 (3.2)	NA	NA	NA	NA	0	12 (92.3%)	1 (7.7%)	NA
	**PBO-LIG 120 mg**	NA	14.4 (1.5)	24.3 (3.7)	NA	9 (75%)	242(200.2)	32.5 (9.0)	16.6 (4.4)	15.9 (4.9)	4.1 (4.1)	NA	NA	NA	NA	0	10 (83.3%)	2 (16.7%)	NA
** Maurer 2019**	**24 mg LIG**	44.1 (14.4)	NA	27.3 (6.3)	31 (72%)	NA	88.2(1262.5)	28.6 (7.1)	12.5 (3.4)	16.1 (4.5)	3.4 (4.0)	0	32 (74%)	8 (19%)	3 (7%)	NA	NA	NA	NA
	**72 mg LIG**	44.3 (12.4)	NA	28.5 (7.1)	61 (73%)	NA	101(157)	31.7 (7.3)	13.6 (4.1)	18.1 (4.3)	3.9 (5.4)	2 (2%)	57 (68%)	20 (24%)	4 (4.6%)	NA	NA	NA	NA
	**240 mg LIG**	42.9 (10.5)	NA	27.9 (6.1)	67 (79%)	NA	74.1(580)	30.3 (7.3)	13.0 (4.3)	17.3 (4.1)	4.1 (5.6)	0	65 (76%)	19 (22%)	1 (1%)	NA	NA	NA	NA
	**300 mg OMA**	41.8 (13.1)	NA	28.1 (6.4)	66 (78%)	NA	86.2(2350)	29.3 (7.9)	12.7 (4.4)	16.6 (4.7)	5.1 (7.5)	4 (5%)	67 (79%)	12 (14%)	1 (1%)	NA	NA	NA	NA
	**placebo**	45.4 (11.2)	NA	27.4 (6.5)	31 (72%)	NA	111.5(217)	31.1 (6.8)	13.6 (4.1)	17.6 (4.1)	3.6 (3.5)	0	31 (72%)	9 (21%)	3 (7%)	NA	NA	NA	NA
	**120 mg LIG single dose**	42.4 (14.5)	NA	27.5 (6.9)	30 (71%)	NA	61.2(222.25)	31.3 (6.9)	13.6 (4.0)	17.8 (4.1)	5.7 (7.5)	2 (5%)	31 (74%)	8 (19%)	1 (2%)	NA	NA	NA	NA

UAS7: Urticaria Activity Score over 7 days**;** HSS7**: **Hives Severity Score over 7 days**; **CSU**: **chronic spontaneous urticaria**; **ISS7**:**Itch severity score over 7 days; BMI**:** Body mass index**; **IgE**: **Immunoglobulin E; NA**: **not applicable

### Quality assessment of the studies included

On using the Cochrane tool for risk of bias 2 on our four included studies, three of them demonstrated some concerns mainly in (bias in measurement of the outcome) and (bias in selection of the reported results) domains while the other one showed low risk of bias. (Supplementary figures. 1 and 2).

### Efficacy outcomes

#### Change from baseline in itch severity score (ISS7) between ligelizumab and placebo at week 12 (adults)

**Fig. 2:**
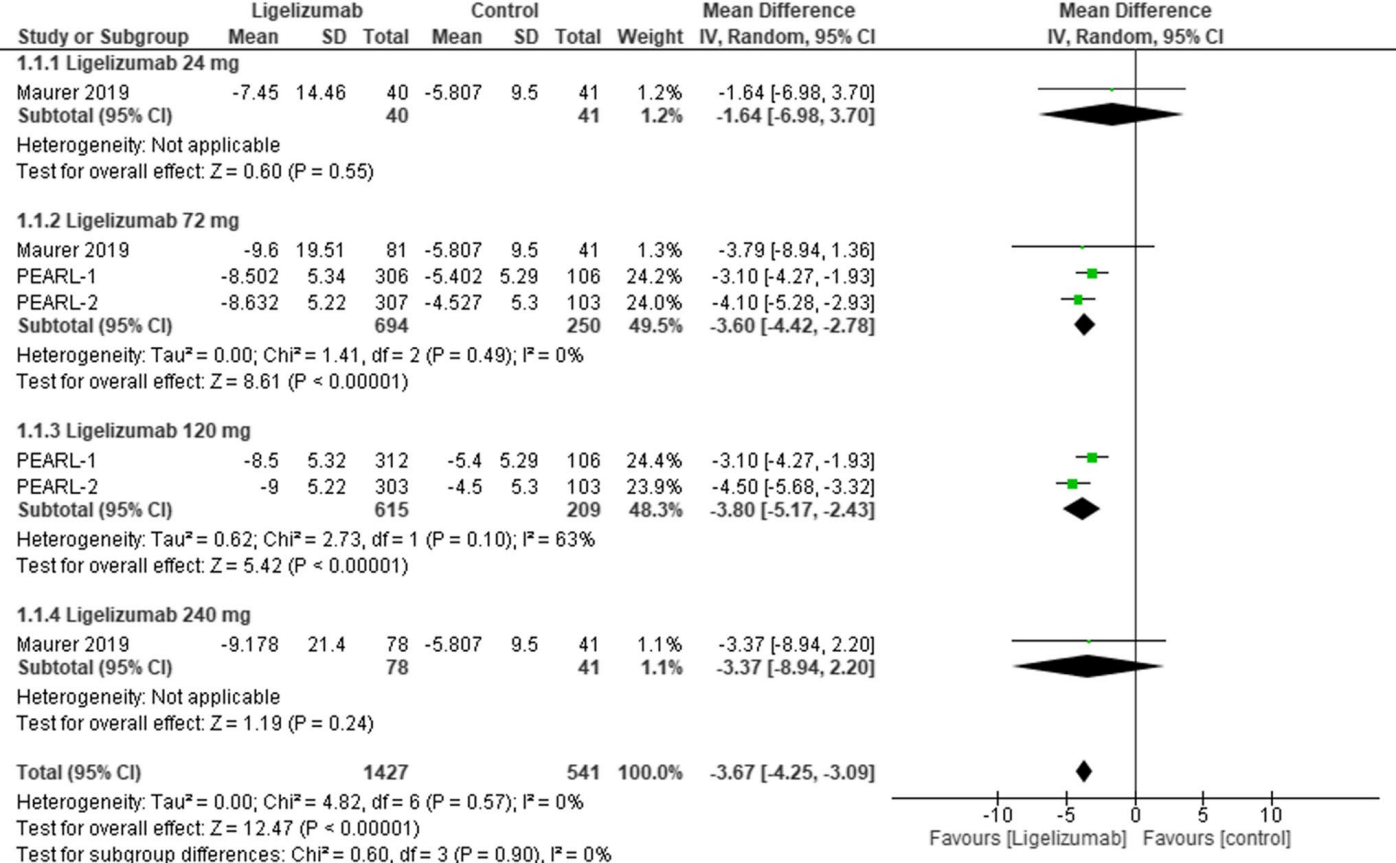
Forest plot comparing (MD) for change from baseline in Itch Severity Score (ISS7) at Week 12 (adults) between Ligelizumab and placebo. The overall effect estimate showed statistically significant difference favoring Ligelizumab

ISS7 was assessed at weak 12 comparing Ligelizumab at different doses with placebo. The overall effect estimates showed statistically significant difference favoring Ligelizumab (MD = −3.67, 95% CI: [−4.25 to −3.09], p < 0.00001) with no overall heterogeneity (I2 = 0%). As for dose subgroups, results were statistically significant favoring Ligelizumab in the 72 mg and 120 mg subgroups (MD = −3.60, 95% CI: [−4.42 to −2.78], p < 0.00001), (MD = −3.80, 95% CI: [−5.17 to −2.43], p < 0.00001) respectively. In contrast, results showed statistically insignificant differences in both 24 mg and 240 mg subgroups (MD = −1.64, 95% CI: [−6.98 to 3.70], *p* = 0.55), (MD = −3.37, 95% CI: [−8.94 to 2.20], *p* = 0.24) respectively.** (**Fig. 2**).**

#### Change from baseline in hives severity score between ligelizumab and placebo (HSS7) at week 12 (adults)

**Fig. 3:**
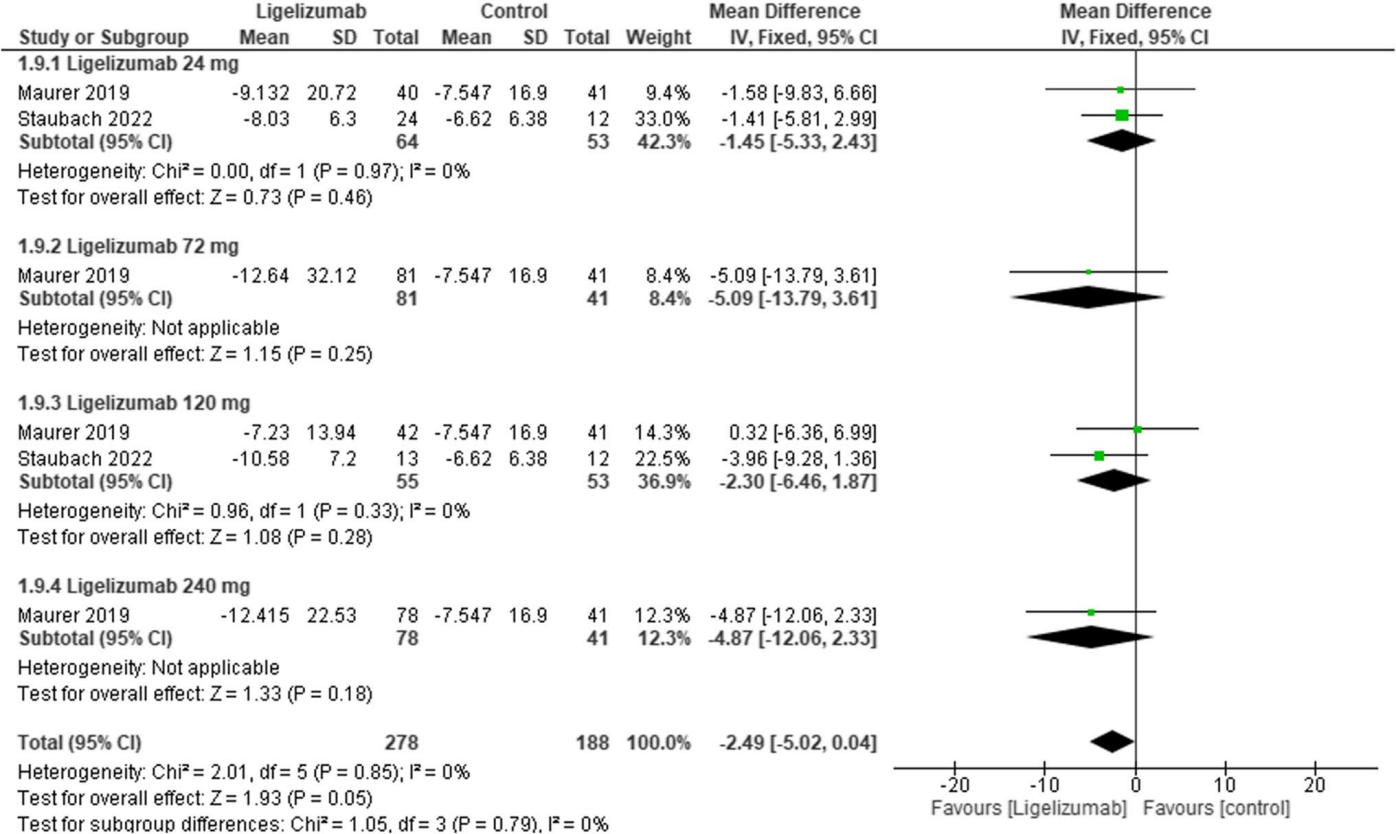
Forest plot comparing (MD) for change from baseline in Hives Severity Score (HSS7) at Week 12 (adults) between Ligelizumab and placebo. The overall effect estimate was statistically insignificant, not favoring either of the two groups

This outcome assessed the change from baseline HSS7 at week 12 in adults comparing Ligelizumab at different doses with placebo. The overall effect estimate was statistically insignificant, not favoring either of the two groups (MD = −2.49, 95% CI: [−5.02 to 0.04], *p* = 0.05) with no overall heterogeneity (I2 = 0%). Moreover, results were statistically insignificant in all dose subgroups, Ligelizumab 24 mg (MD = −1.45,95% CI:[−5.33 to 2.43], *p* = 0.46), Ligelizumab 72 mg (MD = −5.09, 95% CI:[−13.79 to 3.61], *p* = 0.25), Ligelizumab 120 mg (MD = −2.30, 95% CI:[−6.46 to 1.87], *p* = 0.28), Ligelizumab 240 mg (MD = −4.87, 95% CI:[−12.06 to 2.33], *p* = 0.18).**(**Fig. 3**).**

#### Change from baseline in urticaria activity score (UAS7) at week 12 (adults)

**Fig. 4:**
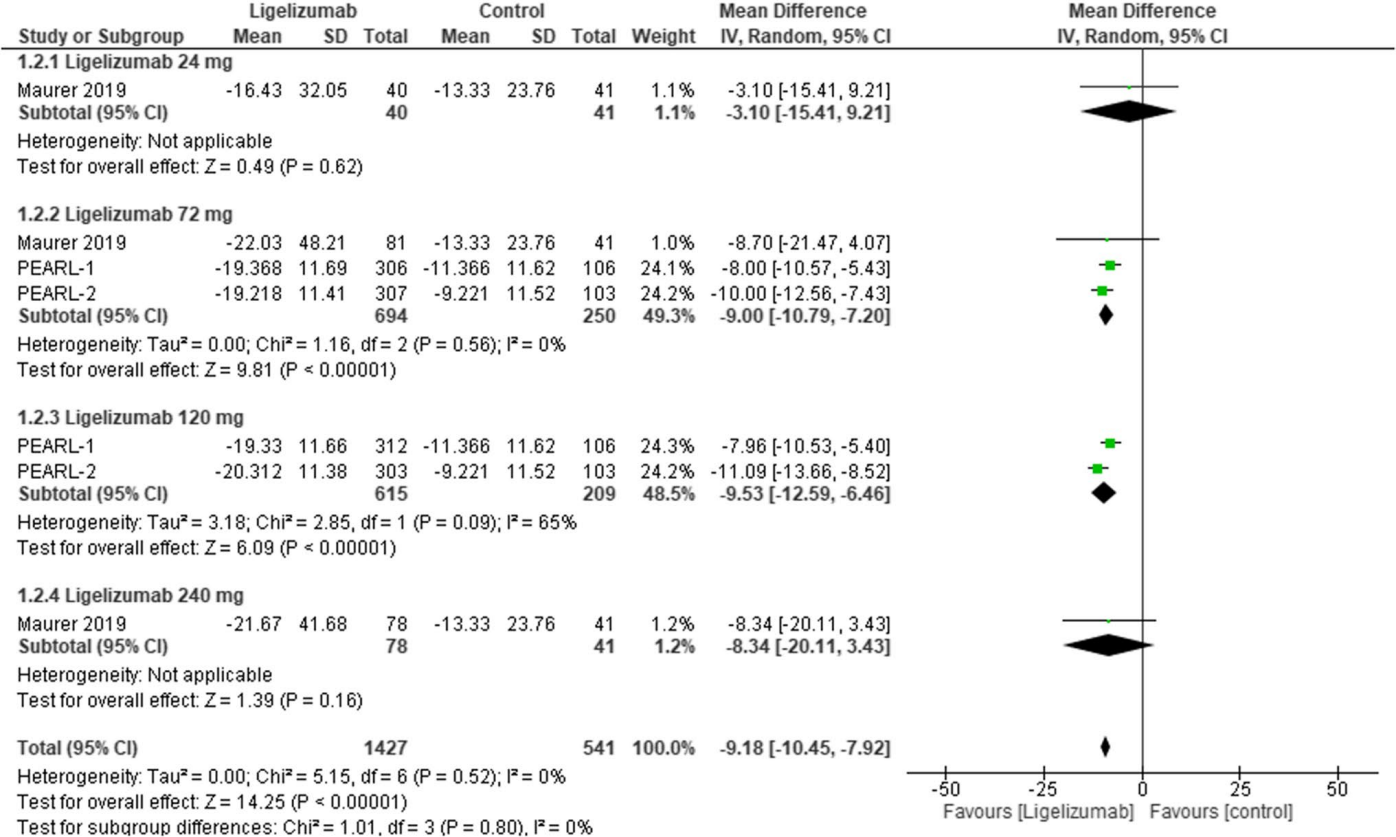
Forest plot comparing (MD) for change from baseline in Urticaria Activity Score (UAS7) at Week 12 (adults) between Ligelizumab and Placebo. The overall effect estimate was statistically significant favoring Ligelizumab

This outcome assessed the change from baseline UAS7 at week 12 in adults comparing Ligelizumab at different doses with placebo. The overall effect estimate was statistically significant favoring Ligelizumab (MD = −9.18, 95% CI: [−10.45 to −7.92], p < 0.00001) with no overall heterogeneity (I2 = 0%). As for dose subgroups, results revealed statistically significant differences favoring Ligelizumab in doses 72 mg and 120 mg as follows; subgroup Ligelizumab 72 mg (MD = −9.00, 95% CI: [−10.79 to −7.20], p < 0.00001) with no heterogeneity, and subgroup Ligelizumab 120 mg (MD = −9.53, 95% CI: [−12.59 to −6.46], p < 0.00001) with moderate heterogeneity (I2 = 65%). While showing statistically insignificant difference in doses 24 mg and 240 mg as follows; subgroup Ligelizumab 24 mg (MD = −3.10, 95% CI: [−15.41 to 9.21], *p* = 0.62), and subgroup Ligelizumab 240 mg (MD = −8.34, 95% CI: [−20.11 to 3.43], *p* = 0.16) (Fig. 4**).**

#### UAS7 response (adults)

**Fig. 5:**
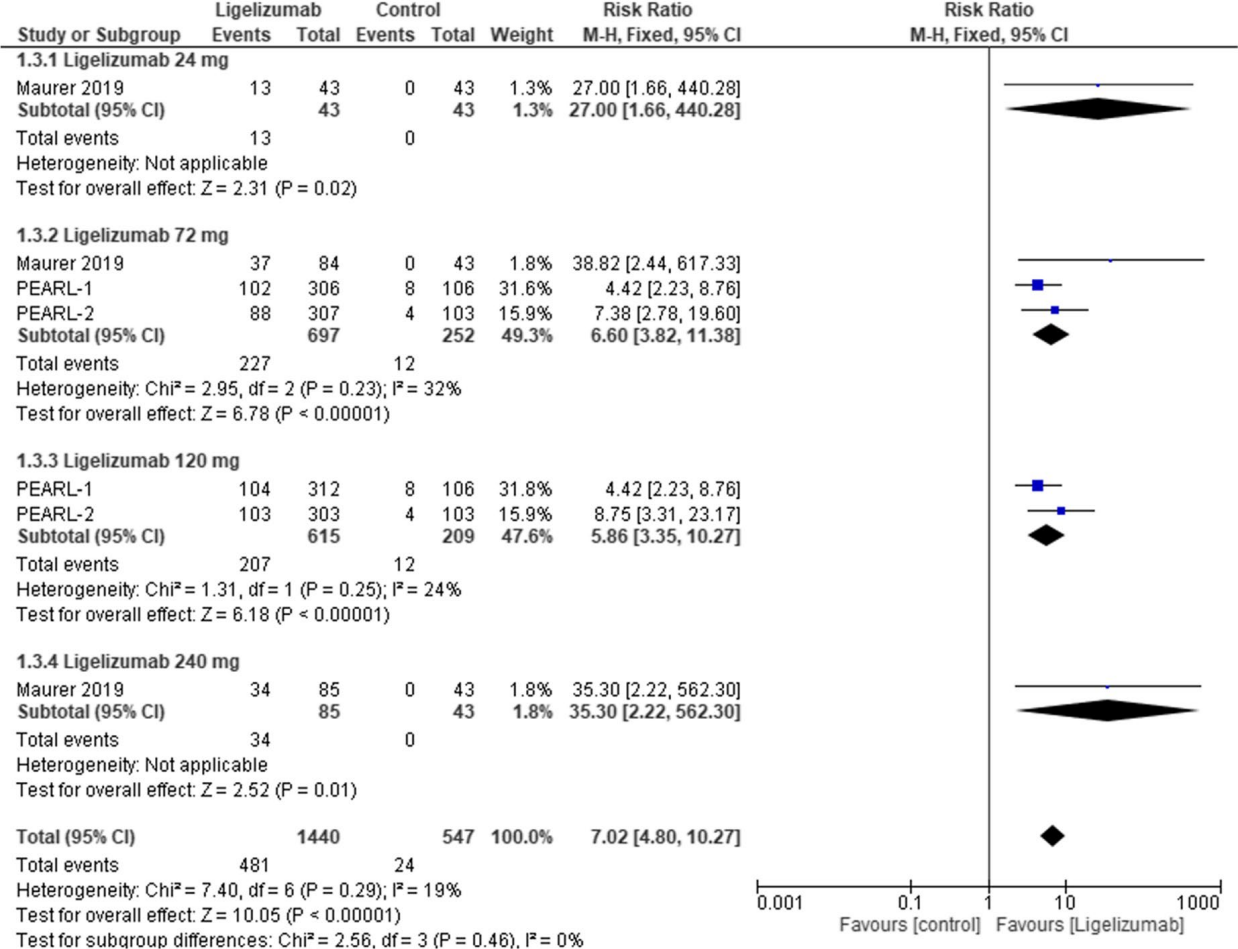
Forest plot comparing (RR) for change in UAS7 Response (adults) between Ligelizumab and Placebo. The overall effect estimate was statistically significant favoring Ligelizumab

This outcome assessed the UAS7 Response in adults to compare Ligelizumab at different doses to placebo. The overall effect estimate was statistically significant favoring Ligelizumab (RR = −7.02, 95% CI: [4.80 to 10.27], p < 0.00001) with no overall heterogeneity (I2 = 0%) between subgroups. Moreover results were statistically significant favoring Ligelizumab in all subgroups, Ligelizumab 24 mg (RR = 27.00, 95% CI: [1.66 to 440.28], *p* = 0.02), Ligelizumab 72 mg (RR = 6.60, 95% CI: [3.28 to 11.38], p < 0.00001) with non-significant mild heterogeneity (I2 = 32%), Ligelizumab 120 mg (RR = 5.86, 95% CI: [3.35 to 10.27], p < 0.00001)with non-significant mild heterogeneity (I2 = 24%), Ligelizumab 240 mg (RR = 35.30, 95% CI: [2.22 to 562.30], *p* = 0.01) with no heterogeneity (I2 = 0%) **(**Fig. 5**).**

#### Change from baseline in urticaria activity score (UAS7) at week 12 (adolescent)

**Fig. 6:**
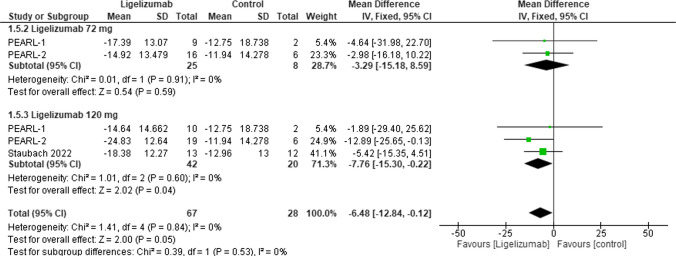
Forest plot comparing (MD) for change from baseline in Urticaria Activity Score (UAS7) at Week 12 (adolescent) between Ligelizumab and Placebo. The overall effect estimate was statistically significant favoring Ligelizumab

This outcome assessed the Change from Baseline in UAS7 in adolescents at week 12 to compare Ligelizumab to Placebo. The overall effect estimate was statistically significant favoring Ligelizumab (MD = −6.48, 95% CI: [−12.84 to −0.12], *p* = 0.05) with no overall heterogeneity (I2 = 0%) between subgroups. As for dose subgroups, results showed statistically insignificant difference in Ligelizumab 72 mg subgroup (MD = −3.29, 95% CI: [−15.18 to 8.59], *p* = 0.59). While showing statistically significant difference in Ligelizumab 120 mg subgroup (MD = −7.76, 95% CI: [−15.30 to −0.22], *p* = 0.04) with no heterogeneity (I2 = 0%) in both subgroups. **(**Fig. 6**).**

#### UAS7 response (adolescent)

**Fig. 7:**
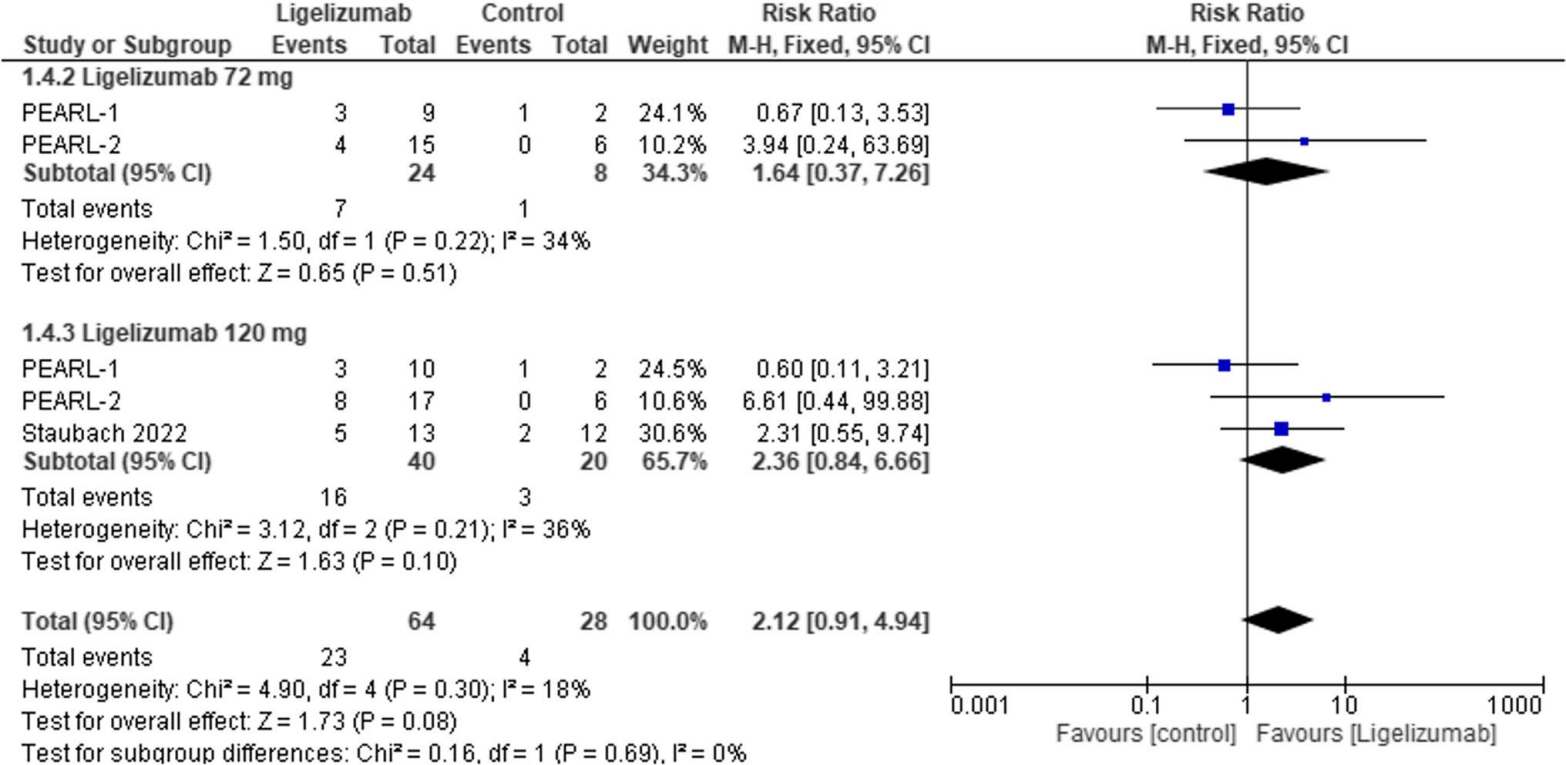
Forest plot comparing (RR) for change in UAS7 Response (adolescent) between Ligelizumab and placebo. The overall effect estimate was statistically insignificant, not favoring either of the to groups

The same outcome (UAS7 Response) was assessed in adolescence to compare Ligelizumab at different doses to placebo. The overall effect estimate was statistically insignificant, not favoring either of the two groups (RR = 2.12, 95% CI: [0.91 to 4.94], *p* = 0.08) with no overall significant heterogeneity (I2 = 18%). Similarly, results were statistically insignificant in all dose subgroups, Ligelizumab 72 mg (RR = 1.64, 95% CI: [0.37 to 7.26], *p* = 0.51), Ligelizumab 120 mg (RR = 2.36, 95% CI: [0.84 to 6.66], *p* = 0.10) with non-significant heterogeneity in either subgroups. **(**Fig. 7**).**

### Safety outcomes

**Fig. 8:**
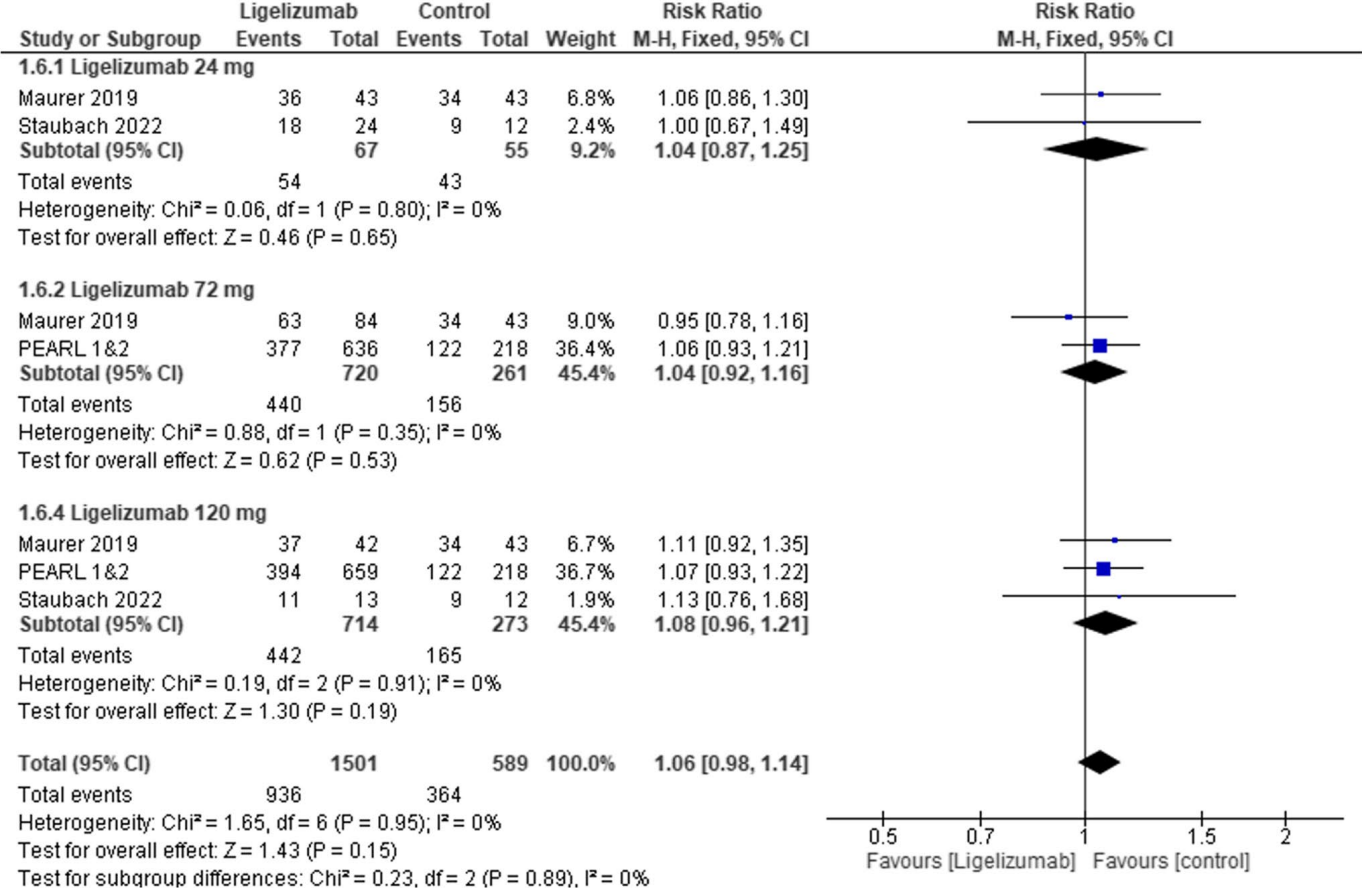
Forest plot comparing (RR) for patients with at least 1 AE between Ligelizumab and Placebo. The overall effect estimate was statistically insignificant, not favoring either of the two groups

**Fig. 9:**
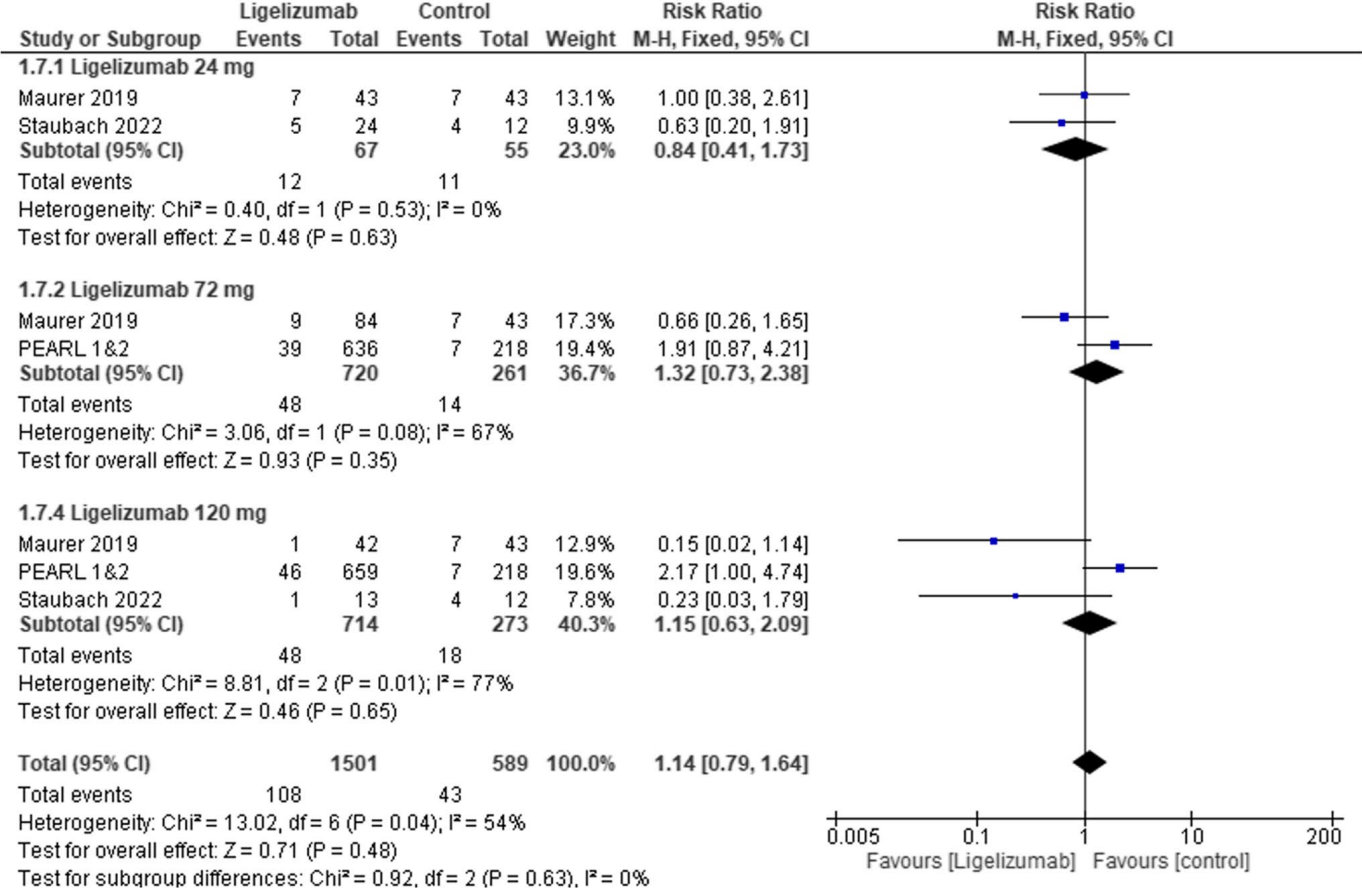
Forest plot comparing (RR) for Headache between Ligelizumab and Placebo. The overall effect estimate was statistically insignificant, not favoring either of the two groups

In TEAEs no statistically significant differences were observed between Ligelizumab 24 mg, or Ligelizumab 72 mg, 120 mg and placebo (RR = 1.04, 95% CI [0.87 to 1.25], *p* = 0.65), (RR = 1.04, 95% CI [0.92 to 1.16], *p* = 0.53), (RR = 1.08, 95% CI [0.96 to 1.21], *p* = 0.19) respectively **(**Fig. 8**).** Similarly in headache no statistically significant difference was observed between Ligelizumab 24 mg, or Ligelizumab 72 mg, 120 mg and placebo (RR = 0.84, 95% CI [0.41 to 1.73], *p* = 0.63), (RR = 1.32, 95% CI [0.73 to 2.38], *p* = 0.35), (RR = 1.15, 95% CI [0.63 to 2.09], *p* = 0.65) respectively **(**Fig. 9).

**Fig. 10:**
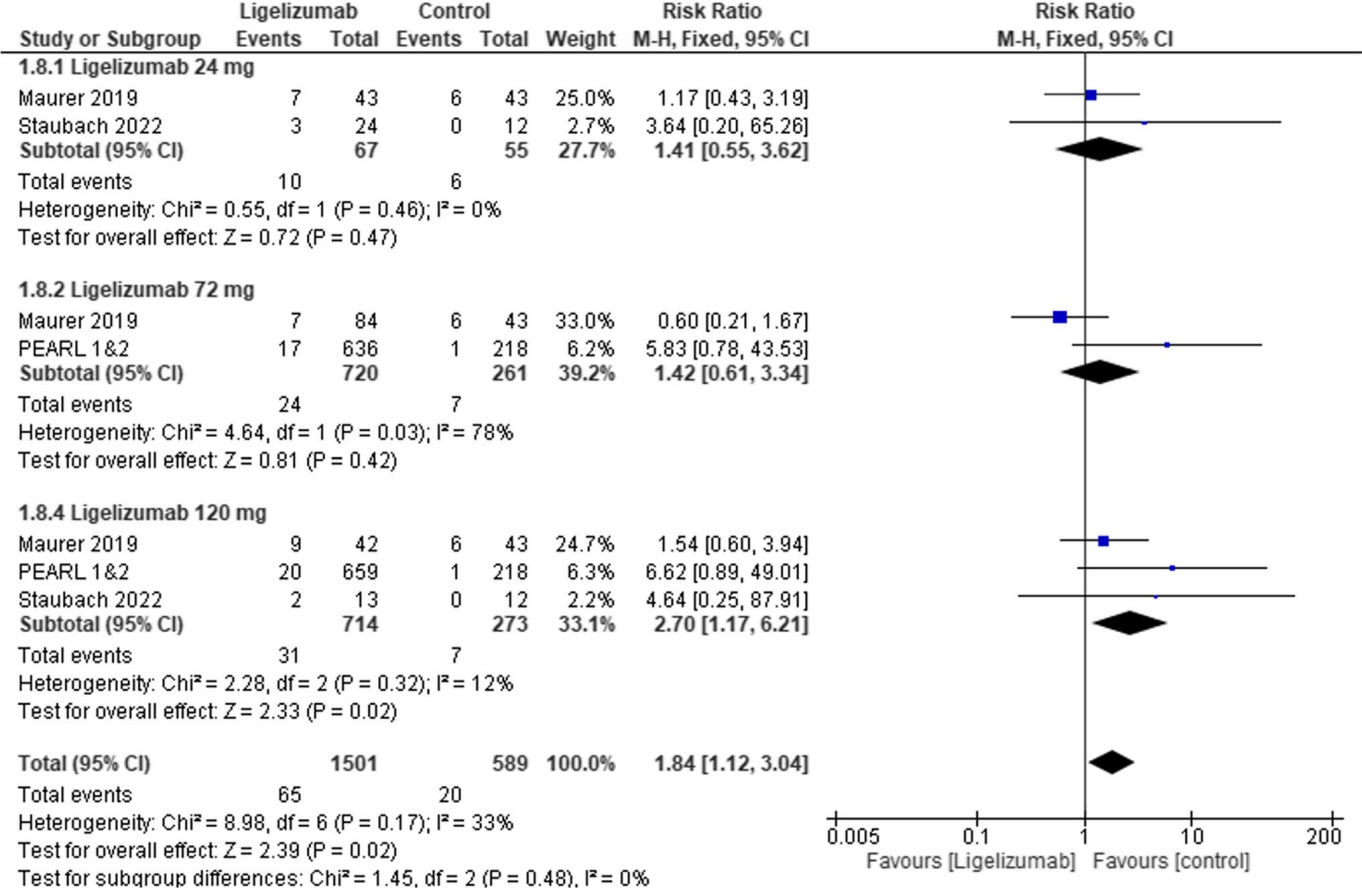
Forest plot comparing (RR) for Upper respiratory tract infection between Ligelizumab and Placebo

Furthermore, in upper respiratory tract infection no statistically significant difference was observed between Ligelizumab 24 mg or Ligelizumab 72 mg, and placebo (RR = 1.41, 95% CI [0.55 to 3.62], *p* = 0.47), (RR = 1.42, 95% CI [0.61 to 3.34], *p* = 0.42), respectively but showed statistically significant difference in subgroup Ligelizumab 120 mg (RR = 2.70, 95% CI [1.17 to 6.21], *p* = 0.02) **(**Fig. 10**).**

### Grade evaluation

The summary of pooled effect estimates including GRADE results, is demonstrated in (Table 3**)**. All primary outcomes showed High level of confidence regarding ISS7 in adults, HSS7 in adults, and UAS7 in adults.

**Table 3:** Summary of overall pooled effect estimates of the primary outcomes

Efficacy Outcomes (follow up)	No. of Studies	No. of Participants	Pooled MD (95% CI)	Overall effect *P*-value	Heterogeneity Chi^2^ *P*-value and *I*^*2*^	Risk of bias	Indirectness	inconsistency	Imprecision	Publication bias	GRADE Evaluation
ISS7 in adults (12 weeks)	3	1968	−3.67 (−4.25 to −3.09)	P < 0.00001	*p* = 0.57, *I*^*2*^ = 0%	No	No	No	No	N/A	**⨁⨁⨁⨁** **High**^**a**^
HSS7 in adults (12 weeks)	2	466	−2.49 (−5.02 to 0.04)	*p* = 0.05	*p* = 0.85, *I*^*2*^ = 0%	No	No	No	No	N/A	**⨁⨁⨁⨁** **High**^**a**^
UAS7 in adults (12 weeks)	3	1968	−9.18 (−10.45 to −7.92)	P < 0.00001	*p* = 0.52, *I*^*2*^ = 0%	No	No	No	No	N/A	**⨁⨁⨁⨁** **High**^**a**^

ISS7: Itch severity score over 7 days; HSS7:Hives Severity Score over 7 days**; **UAS7: Urticaria Activity Score over 7 days**; **MD:Meandifference; CI: confidence interval; MD: mean differenceGRADE Working Group grades of evidenceHigh certainty: we are very confident that the true effect lies close to that of the estimate of the effect.Moderate certainty: we are moderately confident in the effect estimate: the true effect is likely to be close to the estimate of the effect, but there is a possibility that it is substantially different.Low certainty: our confidence in the effect estimate is limited: the true effect may be substantially different from the estimate of the effect.Very low certainty: we have very little confidence in the effect estimate: the true effect is likely to be substantially different from the estimate of effect.Explanations:Note: N/A= Not Applicable because of the small number of included studies. a: There were some concerns in the ROB but not rated down for risk of bias.

## Discussion

Ligelizumab (QGE031) is a novel humanized IgG1 monoclonal antibody that targets and modulates the IgE pathway, which plays an important role in CSU pathogenesis.

Pharmacokinetically (PK), Ligelizumab follows a biexponential distribution with a half-life of approximately 20–23 days, supporting a convenient once-every-four-weeks subcutaneous dosing regimen. Its systemic exposure increases dose-proportionally, and its clearance is modestly influenced by baseline total IgE levels and body weight. Additionally, studies have demonstrated that different subcutaneous delivery systems (e.g., liquid-in-vial versus prefilled syringe), injection volumes, and administration methods (self- vs. healthcare provider-administered) have no clinically relevant effect on the PK profile, thus enhancing the practicality and flexibility of Ligelizumab delivery in real-world settings.

Pharmacodynamically (PD), Ligelizumab leads to rapid and sustained suppression of free IgE in circulation. This in turn downregulates the expression of FcεRI on mast cells and basophils and prevents IgE-mediated activation of these effector cells. Although it also blocks the interaction between IgE and the low-affinity FcεRII/CD23 receptor, this effect appears to be less potent than that of omalizumab. In experimental studies, Ligelizumab induced greater inhibition of allergen-induced skin responses than omalizumab, with skin prick test suppression exceeding 95% compared to ~ 41% with omalizumab six weeks after the last dose.(18,24,25).

Like other therapeutic monoclonal antibodies, Ligelizumab is mainly degraded by proteolytic catabolism into peptides and amino acids, rather than eliminated via membrane transporters or CYP450 enzymes (Ferri et al..(26) It is also cleared through binding to IgE, resulting in faster elimination and a low risk of drug–drug interactions (Arm et al.(18) Furthermore, clinical studies including adults and children with CSU, have not identified any significant pharmacological interactions.

According to the PEARL phase 3 trials, comparing Ligelizumab to omalizumab. Ligelizumab showed superior efficacy over placebo but not over omalizumab, both drugs were effective and well tolerated in patients with antihistamine-refractory CSU. As for efficacy both agents achieved similar improvements in symptoms control and quality of life. Moreover, safety profiles were also similar although Ligelizumab had slightly higher rates of injection site reactions and rare hypersensitivity events (27).

### Efficacy outcomes

Our meta-analysis revealed that Ligelizumab-at specific doses-could be effective in treating symptoms of CSU in both adults and adolescence. For adult patients, both doses (72 mg and 120 mg) showed statistically significant improvement in ISS7 and UAS7 at week 12, while the other two doses (24 mg and 240 mg) failed to demonstrate significant improvement which could be explained by the small number of trials as only Maurer et al.(28) used these doses. However, all four doses showed significant improvement in UAS7 response rate. On the other hand, in adolescence patients Ligelizumab 120 mg showed significant improvement in UAS7 score unlike Ligelizumab 72 mg that failed to. Furthermore, neither of the two doses showed significant improvement in UAS7 response rate. Moreover, in HSS7, none of the used doses showed significant improvement compared to placebo.

Our findings were inconsistent with a network meta-analysis conducted by Nochaiwong et al.(29) that showed that Ligelizumab 72 mg and 240 mg were effective in reducing HSS7 as they included only one study Maurer et al. to examine the drug. A single arm study conducted by Takahashi et al.(30) evaluated the use of Ligelizumab 120 mg in CSU adult patients and found significant improvement in both UAS7 scores and UAS7 response rate at week 12 which is consistent with our results.

Unlike adults, in adolescence patients Ligelizumab 120 mg showed significant reduction in UAS7 score at week 12, but no significant improvement was observed in UAS7 response rate. Moreover Ligelizumab 72 mg showed no significant improvement in either outcome. The difference in efficacy profile between adults and adolescents could be due to the small number of adolescent patients included in our analysis or due to their immune system immaturity. A study conducted by Özçeker et al.(31) suggested that both adolescent and adult showed similar CSU features, but no studies were conducted to evaluate the difference regarding their response to Ligelizumab.

The efficacy profile between Ligelizumab and omalizumab was inconsistent across the trials. Omalizumab 300 mg outperformed Ligelizumab 120 mg in both Maurer et al. and PEARL-1, unlike PEARL-2 that showed opposite results. On the other hand, Ligelizumab 72 mg was superior to omalizumab 300 mg in Maurer et al.(28) unlike PEARL 1 and 2 that showed the opposite confirming the findings of Zhao et al.(32) that indicated that both 72 mg and 120 mg Ligelizumab could be superior to 150 mg (but not 300 mg) omalizumab.

### Safey outcomes

The safety profile was comparable between all doses of Ligelizumab and placebo with no significant difference between the two groups in all safety outcomes except Ligelizumab 120 mg that had significantly higher upper respiratory tract infection rates than placebo. These findings are consistent with Trischler et al.(33) that showed no significant difference between Ligelizumab 240 mg and placebo as well as Gauvreau et al.(19) that showed no significant difference in safety outcomes for Ligelizumab 24, 72 and 240 mg when compared to placebo.

Finally Based on our findings, both 72 mg and 120 mg of SC Ligelizumab every four weeks demonstrated the best balance between efficacy and safety in patients with CSU. Both doses significantly improved clinical outcomes such as ISS7, UAS7 and UAS7 response rate in adults, while 120 mg also showed improvement in adolescents for UAS7 reduction. However, the 120 mg dose was associated with a slightly higher incidence of upper respiratory tract infections, whereas the 72 mg dose maintained an excellent safety profile with minimal adverse effects. Higher doses (e.g., 240 mg) did not show additional clinical benefit and were associated with variability in efficacy outcomes, while lower doses (24 mg) were ineffective. However, these results should be interpreted with caution, as the evidence is limited to short-term outcomes assessed at 12 weeks, with no available data on long-term efficacy or safety. Furthermore, the evidence is derived from four RCTs, which restricts the generalizability of the findings.

### Comparison with previous literature

Two previous meta-analyses have evaluated the efficacy and safety of Ligelizumab in patients with CSU, both yielding results consistent with ours(34,35). However, unlike these studies, we conducted a detailed dose-specific analysis of all available Ligelizumab regimens (24 mg, 72 mg, 120 mg, and 240 mg), comparing each to placebo, and included a broader analysis of adverse events (upper respiratory tract infections and headache) to better understand the drug’s safety profile. Moreover, we performed a comprehensive GRADE assessment to rigorously evaluate the quality of the reported outcomes, thereby strengthening the evidence for future clinical decision-making. Additionally, we analyzed data for adults and adolescents separately, offering novel insights into potential age-related differences. Finally, we thoroughly discussed the PK and PD properties of Ligelizumab, alongside a detailed presentation of baseline patient characteristics across the included studies.

### Strength and limitations

We evaluated different doses of Ligelizumab (24mg, 72 mg, 120 mg and 240 mg) to assess the dose–response relationship and to identify the optimal therapeutic dose to obtain the best outcomes with the least adverse events. Our analysis also included both adult and adolescent patients to understand the impact of the drug on different populations.

Despite these strengths, we also faced some challenges. The limited number of the included studies along with the relatively small population size limited our ability to generalize our findings to the whole population of CSU patients. Also, the baseline total IgE levels varied markedly among the trials; with 181 IU among Ligelizumab 24 mg patients in Staubach et al. to 61.2 IU among Ligelizumab 120 mg patients in Maurer et al. All these limitations may contribute to the high heterogeneity observed among some of the efficacy and safety outcomes hereby limiting the generalizability of our findings. This heterogeneity could also be attributed to the differences in drug doses, patients’ attributes or baseline characteristics, or other reasons among the included trials. Also, follow-up duration in the included studies varies from 6 to 16 months constraining our ability to evaluate the durability of security and viability of Ligelizumab with CSU patients over the long haul.

### Clinical implications and recommendations

Ligelizumab offers a new alternative for both adult and adolescent patients suffering from CSU by providing clinical relief with an acceptable safety profile, thus it can be used as an alternative or in adjunct with omalizumab as it is considered the only available drug for CSU patients to reduce the severity of the disease and enhance the quality of life.

Future research should focus on larger, well-designed clinical trials to confirm our findings and explore the long-term efficacy and safety of Ligelizumab and to decide whether it can be used for long durations with acceptable outcomes. Future trials should also compare Ligelizumab with omalizumab 300 and 150 mg as it is the only approved drug for CSU now. Also, new RCTs should include more adult, adolescent and pediatric patients to examine the effect of the drug on the different populations.

## Conclusion

Our findings suggest that Ligelizumab, at doses 72 and 120 mg, has the potential to be an effective drug for CSU in adults and enhance patients’ quality of life with acceptable safety profile with some concerns regarding Ligelizumab, 120 mg. Nevertheless, future studies should focus on including more patients with longer follow-up periods to address the heterogeneity and to establish the optimal dosing regimen for different populations.

## Supplementary Information

Below is the link to the electronic supplementary material.

ESM 1 (DOCX 337 KB)

## Data Availability

The datasets used and/or analyzed during the current study are available from the corresponding author on reasonable request.
