# Supplementary material for: Efficacy and safety of different doses of ligelizumab in patients with chronic spontaneous urticaria: a systematic review and meta-analysis of randomized controlled trials with GRADE evaluation
Source: Naunyn Schmiedebergs Arch Pharmacol. 2025 Nov 18;399(4):6111–26. doi: 10.1007/s00210-025-04775-8 (PMC13046579; doi:10.1007/s00210-025-04775-8)
Supplement: Supplementary file 1 — (DOCX 337 KB) [file 210_2025_4775_MOESM1_ESM.docx]

**
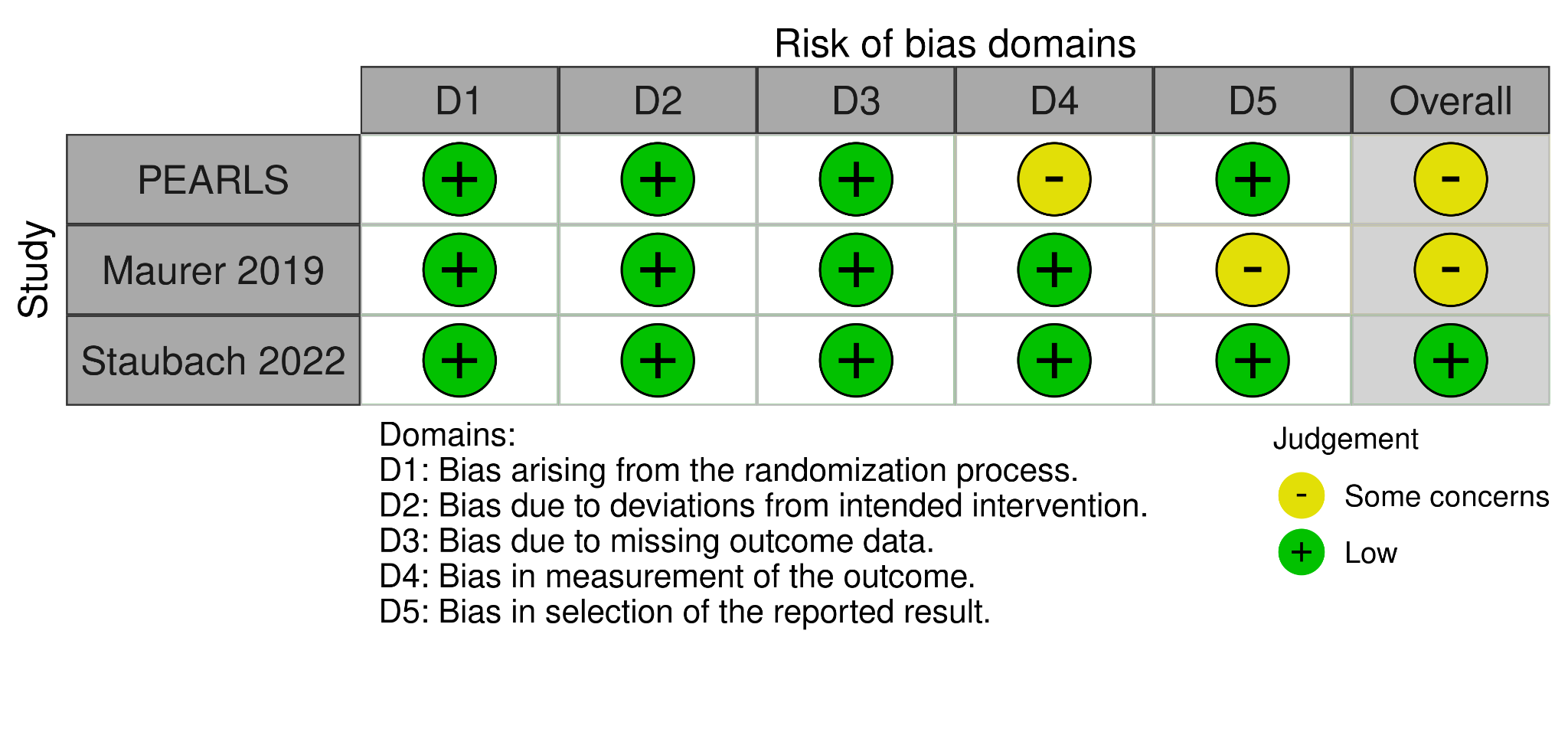
(Supplementary Figure 1).** The bias-risk assessment diagram of the included articles**.**

**
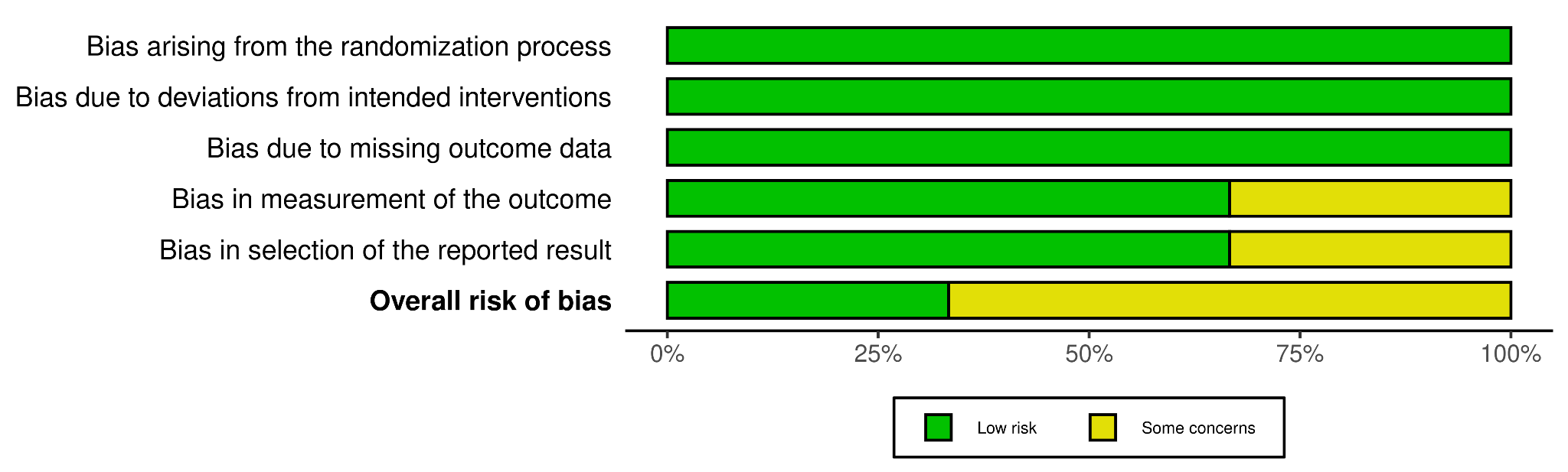
(Supplementary Figure 2).** The bias evaluation bar graph of the included articles.
